# Participatory Ergonomics Intervention to Prevent Work Disability Among Workers with Low Back Pain: A Randomized Clinical Trial in Workplace Setting

**DOI:** 10.1007/s10926-022-10036-9

**Published:** 2022-04-06

**Authors:** Erja Sormunen, Eija Mäenpää-Moilanen, Hilkka Ylisassi, Jarno Turunen, Jouko Remes, Jaro Karppinen, Kari-Pekka Martimo

**Affiliations:** 1 Finnish Institute of Occupational Health, P.O. Box 40, 00032 Työterveyslaitos, Helsinki, Finland; 2 Center for Life Course Health Research, Faculty of Medicine, University of Oulu, Oulu, Finland; 3 Medical Research Center Oulu, University of Oulu and Oulu University Hospital, Oulu, Finland; 4 Ilmarinen Mutual Pension Insurance Company, Helsinki, Finland

**Keywords:** Clinical trial, Occupational health service, Workplace, Low back pain, Ergonomics

## Abstract

*Purpose* In order to support people with low back pain (LBP) to stay at work, work arrangements are regarded important. This study aimed to evaluate the effectiveness of a workplace intervention using a participatory approach on work disability of workers with ongoing or recurrent LBP. *Methods* A total of 107 workers with LBP, with duration of pain for at least two consecutive weeks or recurrent pain of any duration during the last year, were randomized either to the intervention (n = 51) or control group (n = 56). The intervention included arrangements at the workplace, along with individual guidance provided by an occupational physiotherapist (OPT). The randomized intervention study used standard counselling and guidance by an OPT without workplace intervention as a comparison. Surveys were completed at baseline, and 6 and 12 months after baseline. *Results* There were no statistically significant differences between the intervention and control groups on the primary outcome measure, i.e. self-assessed work ability. We found no between-group differences in perceived health, self-assessed work productivity, number of sickness absence days and severity of back pain. However, there were significant positive within-group changes in the intervention group in the intensity of LBP, perceived health and the number of sickness absence days due to LBP. *Conclusion* Workplace arrangements are feasible using participatory ergonomics, but more quantitative and qualitative research is needed on its utilization and effectiveness among workers with LBP.

**Supplementary Information:**

The online version contains supplementary material available at 10.1007/s10926-022-10036-9.

## Introduction

Musculoskeletal disorders (MSDs) are a major threat to work ability and a national health and economic problem in many countries. Low back pain (LBP) is the most common form of MSDs impairing functional capacity. In Finland, the costs of sickness benefit due to back pain were approximately 83 million € in 2020 [1]. However, sickness absence is not the only consequence of LBPs, as musculoskeletal problems cause additional costs through workers’ impaired well-being, as well as reduced work efficiency and, hence, reduced productivity [2, 3].

Occupational physical exposure, for example prolonged standing or walking, repeated lifting and awkward work postures, is associated with back problems [4–6] and multisite pain [7]. However, both LBP experience [8] and multisite pain [7] have multidimensional etiology influenced by biological, physiological and social factors.

From the perspective of functional capacity and work ability, work arrangements and work modifications are regarded important for enabling continuation at work despite musculoskeletal disorders, including LBP [9–12]. It is impossible to unequivocally indicate which work or workplace measures would be the most effective in reducing LBP-related disability. No single work-related measure alone can reduce the impact of LBP on coping with demands at work. Instead, more extensive modifications of work and the work environment, together with ergonomic guidance, have been found to be effective [13, 14]. Among workers with LBP, relatively strong evidence of a link has been observed between workplace interventions and an earlier return to work, improved self-assessed performance, and reduced sickness absence and perceived pain [15].

In previous studies, modifications at the workplace have included the re-organization of work, working time arrangements, improvement of work environment, working postures and movements, and usability of machines and assistive devices [9, 15–18]. Most successful workplace interventions have been conducted when the workers already have musculoskeletal symptoms or disorders that require partial or full-time sickness absence and considerably more investment in rehabilitation [15]. Information is lacking on the effectiveness of workplace interventions conducted at an earlier phase and supporting the work ability of workers at risk of LBP related disability. However, it is known that work-related ergonomic measures are more effective the earlier they are implemented [17, 19].

Participatory ergonomics are commonly used workplace interventions involving workers and employer representatives in developing and implementing arrangements in work and workplace [16, 20]. The main goal is to identify and analyze the problems and possible resources, as well as to develop and implement solutions for improving health and reducing work disability. There are different was to participate, but our focus is on the direct participation in which workplace actors have possibility to influence the decisions regarding work and workplace changes. This randomized controlled study aimed to evaluate the effectiveness of a participatory ergonomics workplace intervention in preventing work disability among workers with LBP using standard occupational physiotherapists’ (OPT) counselling and guidance without workplace intervention as a comparison.

## Methods

### Study Design

**Fig. 1:**
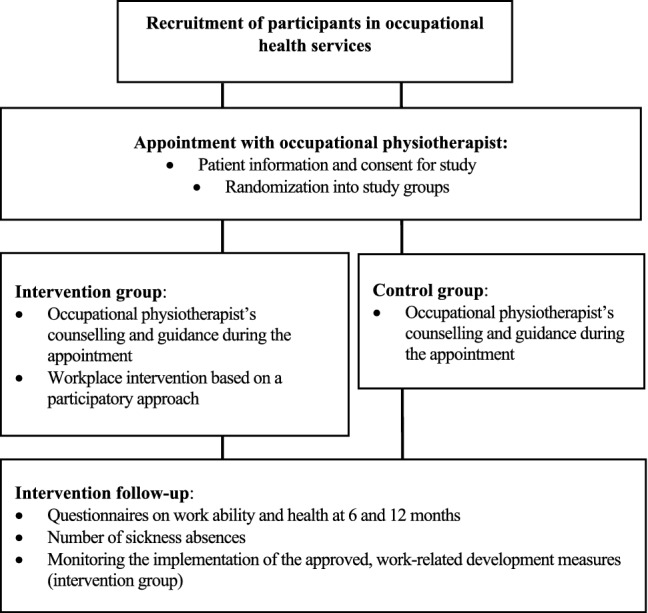
Flowchart of the study design

This randomized, controlled study assessed the effects of ergonomics arrangements on the participants’ work ability comparing workplace intervention using a participatory approach [16, 20] with the standard counselling and guidance by an OPT without workplace intervention. The study design is presented in Fig. 1. The study was registered with ClinicalTrials.gov under the registration ID of NCT03481426.

### Selection and Randomization of Participants

The study was conducted between February 2017 and April 2019 in a total of 18 occupational health service (OHS) units in Finland. We selected the units based on previous research collaboration and an open call through the membership register of the Finnish Association of Physiotherapists in Occupational Health.

The study recruited workers aged 18–65 who had light, moderate or strenuous physical work, and who had suffered LBP, regardless of the intensity or bothersomeness of the pain, (1) with duration of pain for at least two consecutive weeks or (2) recurrent pain of any duration during the last year. Overall, we recruited workers with LBP irrespective of the length of sickness absence due to LBP during the previous year. If the worker had physician-diagnosed LBP, we considered the following back-related diagnoses of the International Classification of Diseases (ICD-10) as described in Table 1.

**Table 1:** Specification of the physician-diagnosed low back pain according to the International Classification of Diseases (ICD-10)

ICD-10 codes	Diagnoses according to the International Classification of Diseases (ICD-10)
M54.4	Lumbago with sciatica
M54.5	Low back pain, unspecified
M54.8	Other dorsalgia
M54.9	Dorsalgia, unspecified
M51.3	Other thoracic, thoracolumbar and lumbosacral intervertebral disc degeneration
S33	Dislocation and sprain of joints and ligaments of lumbar spine and pelvis

The following codes were used among the study participants if the worker had physician-diagnosed low back pain

The individuals selected for the study (hereafter ‘participants’) had to have at least 6 months’ work history in their present job in the beginning of the intervention. Furthermore, the participants’ employment contract had to be operative until his/her 12-months follow-up period used in this study. We excluded no professions or organizations. Participants with active inflammatory spinal disorders such as axial spondyloarthritis, severe nerve root compression, or who were pregnant or undergoing treatment for a serious illness (e.g. cancer, mental illness) were excluded. Work accommodation measures based on a targeted workplace survey i.e., ergonomics workplace survey, conducted by an OPT during the previous 6 months were also considered as an exclusion criterium.

Participants were recruited during occupational physician, nurse and physiotherapist appointments. A worker who met the study inclusion criteria and was interested in participating was referred to the OPT, who described the content and implementation of the study both orally and in writing. The worker was asked for written consent to participate in the study. The OPT contacted by phone the principal investigator (ES), who randomized the worker into either the workplace intervention (intervention group) or the control group.

An Excel table was created for each OHS unit involved in the study, into which the participants were randomized and matched according to their gender into the study groups. The principal investigator randomized the workers according to their order of registration. Participants belonging to the intervention group were asked for permission for the physiotherapist to contact the employer about implementing the workplace intervention.

### Study Intervention

All participants received counselling and guidance from an OPT in their own OHS unit. The participants of both groups were also guaranteed a check-up visit, if required, as well as medical treatment. This was to ensure that each participant received the appropriate medical care and physiotherapy they needed, regardless of which group they were randomized into.

The aim of the intervention was to promote and support the work ability of workers suffering from acute or recurrent LBP through participatory ergonomics and work arrangements implemented at the workplace. The OPT co-ordinated the implementation of participatory ergonomics working method [16, 20]. The worker, employer, and occupational safety and health representative participated in the workplace visit.

The control group participants received counselling and guidance from the OPT. In the control group, no workplace visits or special OHS arrangements were carried out. If, during the study, a workplace visit was seemed necessary for a worker in the control group, this was conducted as part of routine OHS. These changes were recorded and considered when reviewing the results.

### Workplace Intervention

The workplace intervention protocol, based on a participatory ergonomics, involved all the participants i.e., the worker, employer and occupational safety and health representative and an OPT, to attend and influence the process of workplace visit and the work modification outcomes [16, 20]. The participatory protocol assumes that the best knowhow for work development and implementation of work modifications is on the workplace.

The OPTs in the study were trained for the participatory workplace intervention used in the project. The training sessions, separately for each OHS units, were held before the beginning of the study by the researchers (ES, EMM and HY). The 1-h training consisted of the theoretical background of participatory workplace activities and guidance on the implementation and follow-up of the first workplace visit. The workplace visit was divided into three phases:

#### Phase 1

The worker’s work tasks, work arrangements, work environment, working methods, and physical strain on the body were assessed using a structured observation and assessment form [16]. The worker’s work tasks were classified, and their frequency were recorded on the form using four categories: (1) occasionally = e.g. once a week or month; (2) regularly = e.g. few times a week, maybe once a day; (3) often = e.g. several times a day; (4) continuously = e.g. throughout the day, several times an hour. The interference of job performance due to LBP was assessed using five categories: (1) no pain at all; or pain complicates work tasks (2) a bit; (3) somewhat; (4) a lot; or (5) pain prevents working.

The aim was to determine the impact of back pain on job performance and the order of priority of work-targeted development measures. The priority of the measures was determined by multiplying the figure that described the frequency of each work task by the figure that described the effect of LBP on the performance of the specific task (Online Appendix 1).

#### Phase 2

During the workplace visit, the OPT, supervisor, occupational safety representative and worker together identified solutions to the work-related problems. The task of the OPT was to encourage all stakeholders to discuss solutions and work modification measures. The description and implementation method of the solutions were recorded on the observation and assessment form. During the workplace visits, the stakeholders also agreed on and recorded the schedule and responsible persons for the work development measures. If the measure required purchases or changes to work arrangements, these were the responsibility of the employer.

In the study, ergonomics was understood as a broad approach that seeks to modify work, work environment, work methods and tools to suit the worker, considering the entire work process [21, 22]. The workplace interventions targeted the following areas for ergonomic development: (1) work environment and workspaces, (2) work arrangements, (3) tools and other technical solutions, (4) work postures and movements, and (5) other solutions, e.g. supplementary training [23, 24].

#### Phase 3

After the workplace visit, the OPT was responsible for monitoring the implementation of the approved changes by telephone, email or a new workplace visit. Follow-up was organized every 3 months, so that it ended at either 12 months from the start of the intervention or once the agreed measures had been completed.

The completed workplace visits forms and the follow-up forms were sent to the principal investigator, who transferred the data to an Excel spreadsheet for analysis.

### Data Collection and Methods

The questionnaires elicited the participants’ background information (e.g., age, gender, occupation, work history in current job, main form of working time, number of weekly hours); health status; prevalence, severity, and interference of LBP at work; perceived strenuousness of work; and self-assessed work productivity. The questionnaires also elicited the prevalence of work-related physical strain factors [25].

The main response variable of the study was the worker’s self-assessed work ability compared to lifetime best at the start of the intervention, as well as 6 and 12 months after the workplace intervention [26]. The values ranging from number 0 = completely unable to work to number 10 = work ability at its best. Other response variables were perceived health, where the best state of health that the participant could imagine is represented by 100 and the worst by 0 (EQ-5D, EuroQol Group 1990), work productivity [17, 27], self-assessed low back functional capacity [28] and severity of LBP assessed on a visual analogue scale of 0–10 cm [29].

Self-assessed work productivity was elicited using a two-question indicator [17, 27]. We asked the participants to evaluate the impact of LBP on the quantity and quality of their work in comparison to a normal working day. A response value of 10 reflected performance typical of a regular working day, whereas values of 0 to 9 indicated a productivity loss. The decrease in productivity was calculated using the formula: 1 − (quantity/10 × quality/10) × 100%.

We also assessed whether perceived work ability, sickness absence, and the severity and bothersomeness of LBP differed in accordance with the workers’ self-assessed psychosocial stress. To assess the risk factors for LBP-related disability, we used the Start Back Tool, which categorizes those with back pain into low-, moderate-, and high-risk groups [30–33].

Number of sickness absence days due to low back problems, both prescribed by a physician and self-reported, were asked at baseline for the last 3 months, while at the 6- and 12- month follow-up surveys for the last 6 months. The implementation and measures of the participatory workplace intervention were evaluated by the structured observation and assessment form (Online Appendix 1). The study follow-up started after the workplace visit.

### Questionnaires

The participants responded to an initial questionnaire in either electronic or paper form at baseline. The follow-up questionnaires were sent out 6 and 12 months after baseline. Those who did not respond were sent a reminder either by email or post.

The collected data were analyzed at baseline and at the 6-month measurement point by comparing the intervention and control groups. We did the same at the baseline and 12-month measurement points.

### Sample Size

The study considered significant a difference of 10% in the self-assessed work ability [26] and the sickness absence days between the intervention and control groups. We selected a p-value of 0.05 and a power level of 80%. The size of the required study sample was determined to be at least 90 participants for both the intervention and control groups, i.e., 180 participants in total.

### Statistics

The continuous variables were tested using the normally distributed t-test and the non-normally distributed variables using the Wilcoxon test. In the generalized linear multivariate model, in response to difference of 12 to 0 months, the value of the intervention group was compared with that of the control group. The difference between the groups was adjusted for age and gender. For the analysis of the quantitative data, we used the SPSS statistics programme (version 27). The result was statistically significant if p-values < 0.05.

## Results

### Participant Baseline Characteristics

**Fig. 2:**
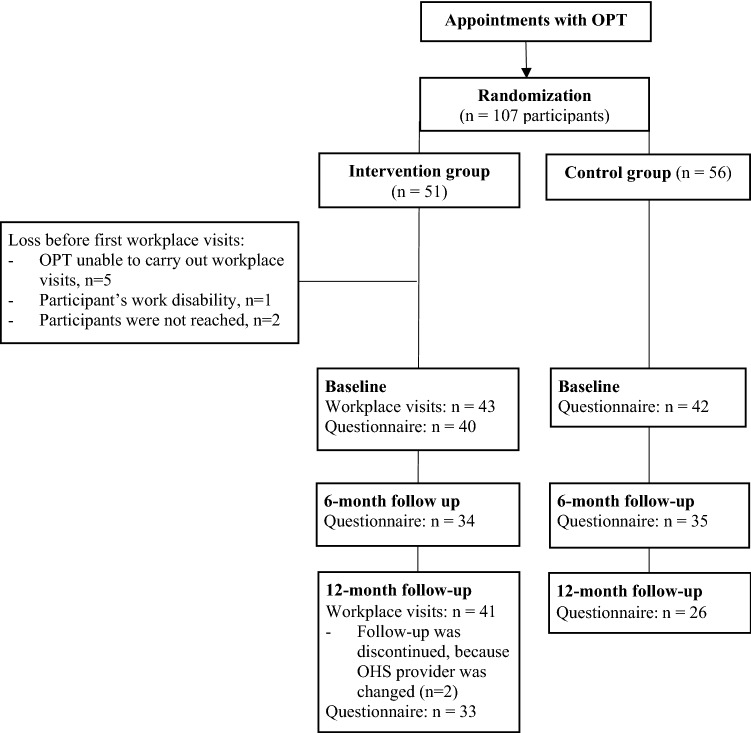
Progression of study and study population during intervention

The OPTs recruited a total of 107 workers with ongoing or recurrent back problems, of whom 51 were randomized into the intervention and 56 into the control group. The initial questionnaire was answered by 40 people in the intervention group and 42 people in the control group. A total of 69 participants responded to the 6-month follow-up questionnaire (34 in the intervention group and 35 in the control group). A total of 59 people responded to the 12-month questionnaire, of whom 33 belonged to the intervention and 26 to the control group. Figure 2 presents the progression of study and study population during the intervention.

The majority (74%) of those recruited for the study were women. There were no significant differences between the study groups on the self-assessed physical workload. Mostly, the participants assessed their own workload as strenuous; 49% in the intervention group and 45% in the control group. Respectively, workload was assessed as light/fairly light by 28% and 35%; and as very strenuous by 23% and 19% of the participants. Table 2 describes the background data of the participants in the intervention and control groups.

**Table 2:** Background information on intervention and control group participants at baseline

Variable	Intervention group (n = 40)	Control group (n = 42)
*Age*; years, Mean (SD)	48 (8)	46 (8)
*Field* by profession; % of participants		
Industry	18	17
Social and health care sector	40	52
Early childhood education and teaching	28	17
Other (Office, sanitation)	15	14
*Prevalence of low back pain*; % of participants	74	77
*Bothersomeness of low back pain in current work*; % of participants		
None at all	5	9
I can perform my work, but it causes symptoms	28	35
I sometimes or often have to reduce the pace of work or change the way in which I work	62	49
Because of my illness, I think I could only cope with part-time work	3	0
I think I’m completely incapable of work	3	7
*Number of sickness absence days in last three months*; Mean (SD)	6 (12)	3 (7)
*Self-assessed work ability in terms of physical demands of work*; % of the participants		
Very good or fairly good	38	48
Moderate	49	43
Fairly poor or very poor	13	10

The results are % shares or averages (standard deviation)

### Effects of Participatory Ergonomics Intervention on the Prevention of Work Disability

#### Work Ability, Perceived Health and Productivity Loss

The groups’ self-rated work ability compared to lifetime best did not differ significantly at baseline and did not change significantly in either group nor between the groups during the whole follow-up. Table 3 presents the mean difference in work ability from baseline to 12-month follow-up.

**Table 3:** Differences between the intervention and the control group in self-rated work ability, perceived health, severity of low back pain and productivity loss during the 12-months follow-up

Variable	Baseline	12-month	Difference between baseline and 12-month	p-value (within group)	Standardized difference	p-value (between groups)
*Work ability compared to lifetime best*						
Intervention group (n = 30)	7.1 (1.8)	7.2 (1.9)	0.13 (2.1)	p = 0.661	0.05	p = 0.932
∙ Control group (n = 23)	8.0 (1.3)	8.2 (1.2)	0.13 (1.1)	p = 0.651	Control	
*Self-assessed state of health*						
Intervention group (n = 31)	53.4 (29.3)	65.3 (29.1)	11.9 (32.4)	p = 0.018	12.3	p = 0.128
Control group (n = 25)	70.5 (24.3)	71.2 (23.3)	0.7 (22.1)	p = 0.727	Control	
*Severity of low back pain in last week*						
Intervention group (n = 31)	5.8 (2.6)	3.1 (2.4)	− 2.7 (2.9)	p < 0.001	− 1.69	p = 0.057
Control group (n = 24)	4.5 (2.8)	3.3 (2.6)	− 1.2 (3.0)	p = 0.087	Control	
*Productivity loss*						
Intervention group (n = 29)	27.9 (27.5)	18.9 (25.3)	− 9.0 (29.4)	p = 0.121	− 6.88	p = 0.352
Control group (n = 24)	23.0 (21.5)	19.6 (21.1)	− 3.3 (20)	p = 0.513	Control	

Mean (standard error). Between groups differences are standardized with age and gender

At baseline, participants in the control group rated their health as better than those in the intervention group, who experienced significantly greater improvement (p < 0.05) to their state of health during the12-month follow-up than those in the control group. However, there were no significant differences between these two groups (Table 3).

In both groups, work productivity was perceived as lower than usual due to LBP. At baseline, the self-assessed work productivity loss of the intervention group was 27.9% and that of the control group 23%. In the intervention group, we observed clear improvement in the self-assessed work productivity between baseline and 12-month follow-up, but there was no statistically significant difference between the groups (Table 3).

#### Low Back Pain Outcomes

At baseline, 74% of the intervention group and 77% of the control group experienced LBP. During the follow-up, the prevalence of LBP significantly decreased (p < 0.05) in both groups, with 41% in the intervention group and 40% in the control group experiencing LBP at the end of follow-up (12 months). There was no statistically significant difference between the groups. The bothersomeness of LBP decreased significantly within the groups during follow-up (p < 0.05), but the difference between the groups was not statistically significant. At baseline, the severity of LBP was higher in the intervention group than in the control group. During follow-up, the severity of back pain decreased significantly in the intervention group (p < 0.001) between baseline and 6-month follow-up and 12-month follow-up (Table 3). In contrast, in the control group, the change in intensity of LBP did not differ significantly between baseline and 12-month follow-up. The difference between the groups, in terms of the severity of LBP, was almost significant in comparison with the beginning and the end of the intervention (p = 0.057).

At baseline, 5% of the participants in the intervention group reported that LBP did not impede their current work at all. At 12-month follow-up, the corresponding figure was 19%. A similar trend emerged in the control group, in which 9% of the participants felt at baseline that their pain did not impede their work at all, whereas this figure was 23% at the end of the intervention. In the intervention group, 42% reported at baseline that they had to ‘sometimes or often reduce the pace of work or change the way in which they work’ because of LBP. At the end of the intervention, only 34% of the respondents felt this way. In the control group, the corresponding figures were 49% at baseline and 54% at the end of the intervention.

#### Sickness Absence and Start BackTool -Outcomes

In both groups, the number of sickness absence days due to LBP decreased between the start and end of the intervention. Within the intervention group, the change was significant (p < 0.05), as absence days decreased from the initial average of 6 days (during the last 3 months before the initial questionnaire) to 2 days (during the last 6 months before the final questionnaire). Correspondingly, in the control group, the number of absence days decreased from an average of three days to one day. There was no statistically significant difference between the groups.

The response variables in the study were also examined in terms of the psychosocial factors (Table 4). The results showed that participants belonging to the high-risk group of the Start Back Tool (n = 10) experienced more severe LBP at baseline, and they had lower self-assessed work ability and more absences from work per se than workers at a low/moderate risk. The 12-month follow-up of workers belonging to the high-risk group showed that the workplace intervention was a more effective measure than control care for reducing their severity of LBP (p < 0.001), when compared to the workers at a low/moderate risk. In contrast, the intervention had no significant impact on self-assessed work ability, work productivity or sickness absence days.

**Table 4:** Examination of response variables of study by risk groups based on Start Back Tool at baseline

	High-risk group (n = 10)	Low/moderate -risk group (n = 73)	
	Mean (SD)	Mean (SD)	Mann–Whitney test
Back pain intensity	8.3 (1.1)	4.6 (2.4)	p < 0.001
Work ability	6.2 (1.7)	7.7 (1.6)	p = 0.007
Reduction in work productivity (%)	23.8 (23.6)	35.0 (25.3)	p = 0.170
Sick leave (self-reported 3 months) at baseline	13.2 (20.3)	5.3 (12.6)	p < 0.001

### Solutions to Problems Identified in Workplace Intervention

The participants of the workplace visit were an OPT, a worker with LBP and his/her supervisor, as well as an occupational safety and health representative. Most (63%) of the workplace visits were conducted in co-operation with all the above-mentioned parties. Eight workplace visits (19%) were conducted by only the OPT and the worker, and six (14%) of the workplace visits involved all parties except for the occupational safety and health representative. Two workplace visits (4%) included an occupational health and safety representative in addition to the worker and the OPT.

Based on the initial questionnaire, the participants both in the intervention (15%) and in the control group (28%) reported that there already have been implemented some changes to their work tasks due to LBP before the intervention study. However, the changes identified by the participants were not specified according to the content, implementation time and context. During the intervention, no work-related changes occurred during the follow-up in the control group, whereas in the intervention group a significantly higher number of participants (47%) reported work-related changes at the end of the follow-up. The differences were significant between the study groups (p < 0.001) and within the intervention group (p < 0.05).

Table 5 describes the jointly developed solutions to strenuous work tasks and problems at work. A total of 345 proposed solutions were identified in the workplace intervention forms (43 participants). One third (32%) of all the recorded ergonomics solutions were related to the worker’s physical exertion at work i.e., ‘Work posture and movement’ category. Another 32% of the solutions were classified under the ‘Tools and technical solutions’ category. Nineteen per cent of the solutions were in the ‘Work arrangement’ category. In terms of figures, the least number of solutions were reported for the modifications to work environment and workspaces (8%), treatment of physical condition and pain (4%) and supplementary training (5%). A given problem may have several solutions (Table 5).

**Table 5:** Classification of ergonomics solutions developed during workplace survey and examples of ergonomics measures in different categories

	Work environment and workspaces	Work arrangements	Tools and technical solutions	Work posture and work movements	Treatment of the worker’s physical condition or pain	Further training
Number of solutions and % shares	27 (8%)	65 (19%)	111 (32%)	112 (32%)	13 (4%)	17 (5%)
Examples of solutions for this area	Improvement to loader’s chassis evenness Removing one shelf block enables the building of wider, proper steps to the upper corridor (present access by ladder) Tidy-up of workspace, getting rid of extra items	Scheduling phone times for customers Work breaks Increasing pair work Downloading a circular calendar for planning and scheduling work	Standing desk Flat mop with an adjustable handle to reduce stretching arms More effective servicing of forklift seats Patient lifters and transfer sheets and sliding sheets	Ergonomic way of working; no back rotation or bending Taking breaks from sitting, occasionally standing at work Counter movements of back after a work phase	Therapeutic exercises Use of a back-support belt if necessary	Repeat training in work postures and assistive devices Ergonomics training (e.g. Ergonomic Patient Handling Card®

The figures represent the number and % shares. A total of 345 ergonomics solutions were recorded

The schedule for implementing the ergonomics solutions was decided based on the form filled during the workplace intervention. The total number of solutions was 345 and the implementation schedule was recorded for 187 solutions, of which 15% (28 solutions) were implemented immediately during the workplace survey. Most of the solutions, 59% (110 solutions), were implemented within 3 months of the workplace survey. One quarter (26%) of the solutions were implemented more than 3 months after the workplace survey, however within the follow-up time. The implementation schedule for the other 158 solutions was not recorded clearly and the implementation schedule could not be assessed.

## Discussion

The aim of this study was to identify the effectiveness of a workplace intervention using a participatory approach on work disability prevention of workers with ongoing or recurrent LBP. The intervention included arrangements at the workplace, along with individual guidance provided by an OPT. The randomized intervention study used standard OPT counselling and guidance without workplace intervention as a comparison. We also examined the implementation and measures of the participatory ergonomics intervention.

The intervention, implemented according to the study protocol, had no statistically significant impact on the primary outcome measure, i.e. work ability. No statistically significant changes occurred in self-assessed work ability scores of either group during the 12-month follow-up. Furthermore, there were no significant differences between the intervention and control groups neither on the perceived health, self-assessed work productivity, number of sickness absence days and severity of back pain.

### Effectiveness of Workplace Interventions

Work arrangements and modifications are significant measures to optimize workload for employees with reduced work ability and to enable their staying at work and return to work after disability period [10]. There are promising results and evidence of a link between workplace interventions and earlier return to work of workers with musculoskeletal disorders e.g. LBP after a period of work disability, improved self-assessed functional capacity, reduced sickness absence, and reduced perceived pain [15, 34].

However, our findings are in accordance with other randomized controlled trials that participatory ergonomics do not have explicit benefit on reducing musculoskeletal pain nor improving work ability. There is still no unequivocal evidence that workplace interventions or ergonomic measures are effective in promoting health and work ability of people with musculoskeletal problems [12, 19, 35, 36]. Successful implementation of participatory ergonomics interventions, as well as other measures to modify work, requires commitment at all levels, for example from employers and workers, and other stakeholders participating in the processes.

Although the present participatory workplace intervention, combined with OPT guidance, did not prove to be a more effective than counselling and guidance in the control group, there were statistically significant positive within-group (but not between-group) change in intensity of LBP and perceived health. At the beginning of the intervention, LBP was associated with a decrease in self-assessed work productivity in both groups. Although work productivity appeared to improve more in the intervention group than in the control group during follow-up, no statistically significant difference was found between the groups. Therefore, the present study contrasts with the studies concluding that work-targeted ergonomic measures are effective in preventing and restoring self-assessed productivity [17] and reduce sickness absence [9, 16, 37] among employees with musculoskeletal problems. Productivity, sickness absence and related costs remain as one of the main drivers for workplaces to conduct interventions preventing musculoskeletal disorders. However, the evidence on the economic results of such interventions is limited [38], suggesting there is a demand for economic evaluation of interventions.

### Ergonomics Solutions

Work ability/disability is a sum of inter-related factors, including physical, psychological and organizational characteristics. There has been no clear consensus on the most appropriate interventions to improve coping with LBP at work. However, recent multicomponent interventions, with a range of biopsychosocial factors, are regarded effective in reducing sickness absence and promoting return to work [19, 39].

The participative workplace visits in the present intervention group identified the strain factors and problems at work for which the participants planned corrective measures and ergonomic solutions. More than a third of the ergonomic solutions focused on improving work postures and movements, and on the use of existing or new tools and assistive devices. The number of solutions concerning the work environment and work arrangements was smaller. Previous ergonomic interventions have also had a similar distribution [15, 17, 40]. It is difficult to evaluate whether work postures and strenuousness of work methods were given more weight in the work development solutions than work environment and work arrangement modifications, or whether the workplaces really had fewer problems in the last-mentioned ergonomics contents.

From the viewpoint of fluency of work and occupational safety, it is important that a broad ergonomics perspective is applied to work and working conditions when developing them, with an aim to modify work, the work environment, work methods and work tools to suit the worker, taking into account the whole work process [21, 22]. In the present study, the intervention and control groups were comparable with respect to the fields of professions, thus enhancing the generalizability of the results.

Lin et al. [41] identified eleven recommendations for musculoskeletal pain care: ensure care is patient centred, screen for red flag conditions, assess psychosocial factors, use radiological imaging selectively, undertake a physical examination, monitor patient progress, provide education/information, address physical activity/exercise, use manual therapy only as an adjunct to other treatments, offer high-quality non-surgical care prior to surgery and try to keep patients at work. Our intervention is consistent with these recommendations. In both groups of our study, individual guidance was given by an OPT for each participant. The content of the guidance was not strictly defined nor analysed at the end of intervention, but it was performed according to their own OHS practice. The individual guidance can be regarded as a significant factor for promoting work ability. Physical exercise programmes, such as resistance training and stretching programmes, have been found to be effective in preventing and managing musculoskeletal problems [34, 42]. From the clinical point of view, this could be noticed as a potential measure for work ability promotion, together with work modifications.

Cochrane et al. [19] expresses the need to identify and target the interventions especially to those who are at a high risk for disability. The review by van Erp et al. [39] showed that interventions with clear focus on psychosocial factors, i.e. understanding pain, coping strategies for unhelpful thoughts and goal settings, are beneficial for persons with chronic LBP. Such beneficial biopsychosocial factors can also be found in our protocol: (1) workplace solutions are applicable to participants’ individual context, (2) the learned information is conceivable into practice and (3) support from OHS and own workplace on how to implement the solutions in daily work. The findings of Cochrane et al. [19] and van Erp et al. [39] can be regarded consistent with our finding that participants with high psychosocial risk had more severe LBP and lower self-assessed work ability. The present workplace intervention was regarded more effective in reducing severity of LBP for the participants belonging to the high-risk group of Start Back Tool than for the workers belonging to low- or moderate-risk groups.

Timing of the intervention and implementing work modifications has been accepted as important to promote return to work and to reduce sickness absence [19, 43]. In the present study, most of the ergonomic solutions of the workplace intervention were implemented within 3 months of the first workplace survey. However, almost half of the ergonomic solutions’ implementation schedules remained unclear. In the survey data, on average, half of the intervention group members identified changes in their own work or work tasks during follow-up. However, almost half of the intervention group members did not feel that their work had undergone any changes since the first workplace survey. This somewhat contradictory result, compared to the implemented workplace surveys, raises the question of whether work development measures had actually been put into practice at all, which would reduce the effectiveness of the intervention. The monitoring of guidance and counselling at the workplace should be more systematically considered and the implementation of development measures ensured also in clinical work in OHS.

### Strengths and Limitations of the Study

The strength of the study was the implementation of a challenging intervention setting in a real work environment. The study tested a new model and tool for assessing and resolving work tasks and problems related to low back pain. The novelty value of our study was that the intervention was implemented in co-operation with OHS, the workplace and occupational safety and health. The participatory approach was achieved in most of the workplace visits. There were only few cases when the workplace visit was conducted by only the worker and the OPT. Another strength was that the intervention model could be used both at an early stage, before long period of absence from work, and at later stage with prolonged sickness absence. It is however known that the earlier work-related development measures are implemented, the more significant they are for workers’ health and continuation at work [17, 19, 43].

Lastly, participatory approach may benefit more rapid procurement of work tools or technical solutions and changes in work processes and lighten the physical workload. These changes can, in the longer term, enhance work ability and reduce the risk for LBP. The positive trends of participative workplace interventions will encourage OHS, workplaces and occupational safety and health actors to co-operate in evaluating work challenges and workload factors, and in work development processes. In addition to examining individuals, group-based evaluation, and development processes from the perspective of the work community would be an interesting topic for further research.

There are also limitations in this study. Firstly, the small size of the groups may be one reason for the non-significant differences. We achieved just over half of the size of the required study sample, 107 participants in total. The OPTs were not asked to keep a record of those who refused to participate the study. Also, it is difficult to assess how well the participants included in the study represented workers with LBP seeking care in OHS. We may, however, speculate that non-participants differed from participants in baseline characteristics thus creating selection bias. Recruitment challenges forced us to change protocol in the middle of the study. We had to abandon the original goal of implementing the intervention only for participants who had sickness absences due to LBP of up to 2 weeks in the preceding year. The change of the protocol increased the heterogeneity of the groups, making it more difficult to demonstrate statistical significance. Differences between the study groups might have been in favor with the intervention, if the original recruitment plan could have been followed, because the study populations would have been more homogenous with respect to LBP history.

Furthermore, demonstrating the effectiveness of workplace interventions in such settings is challenging. Even though the OPTs involved in the study were trained in the use of the model, their practices of the participative method varied. This may have increased variation between the study cases. It is also impossible to assess the effect of the natural healing process associated with back problems. Furthermore, the between-group differences of the intervention may be attenuated by the fact that there may have been some statutory activities of OHS in work tasks to promote wellbeing at work, in the control group as well.

## Conclusion

Although there were no statistically significant differences between the groups, we observed positive trends within the intervention group in, for example, the severity of LBP, perceived health status, and number of sickness absence days. Especially participants scoring high in Start Back Tool on psychosocial risk factors of work disability showed to benefit from the intervention.

More research is needed on the utilization of participatory workplace measures and the evaluation of their effectiveness before participatory ergonomics can be justified to be used among workers with LBP to support their work ability. Qualitative data could give useful information of the intervention practices among the participants and lead to explanations of the current findings.

## Supplementary Information

Below is the link to the electronic supplementary material.

Supplementary file1 (PDF 11 kb)

## Data Availability

The collected data and material (background information, data of the questionnaires and workplace arrangements) is stored in Finnish Institute of Occupational Health (FIOH) in accordance with good research practice. The research material is maintained on FIOH’s network drive and in the locked room that can only be accessed by the project researchers.

## Code Availability

The material will be archived in accordance with the instructions of the FIOH for 5 years, until 31st August 2023, after that the material is discarded. Material and data are not publicly available to preserve individual’s privacy under the Ethics approval of the present study.
