# Supplementary material for: Participatory Ergonomics Intervention to Prevent Work Disability Among Workers with Low Back Pain: A Randomized Clinical Trial in Workplace Setting
Source: J Occup Rehabil. 2022 Apr 6;32(4):731–42. doi: 10.1007/s10926-022-10036-9 (PMC9668957; doi:10.1007/s10926-022-10036-9)
Supplement: Supplementary file 1 — Supplementary file1 (PDF 11 kb) [file 10926_2022_10036_MOESM1_ESM.pdf]

Appendix 1. Observation and assessment form

Employee (name and professional title) ____
Workplace/employer ____
Participants in the workplace visit____

**List your usual work tasks**	**How often the work task (in question) occurs?**<br><br>**4 = continuously** *(e.g. throughout the day, several time an hour)*<br>**3 = often** *(e.g. several times a day)*<br>**2 = regularly** *(e.s. few times a week, maybe once a day)*<br>**1 = occasionally** *(e.g. once a week or month)*	**How much low back pain (LBP) interferences the job performance?**<br><br>**4 = pain prevents working**<br>**3 = pain complicates work tasks, a lot**<br>**2 = pain complicates work tasks, somewhat**<br>**1 = pain complicates work tasks, a bit**<br>**0 = no pain at all**	**Order of priority of work-targeted development measures:**<br><br>The priority of the measures was determined by multiplying the figure that described the frequency of work tasks by the figure that described the effect of LBP on the performance of the specific task

Appendix 1. Observation and assessment form

Issue that causes problems in the work task	Description of the solutions and work development measures	Viability	Person in charge and timetable	Follow-up	Finished
Write here the work tasks that cause problems according to the order of priority; the higher priority number the more important it is to solve that problem.<br><br>- What is the problem?<br>- What causes problem?	Development measures can be targeted into the following ergonomic items:<br>1. work environment and workspaces,<br>2. work arrangements,<br>3. tools and other technical solutions,<br>4. worker physical exertion at work (work postures and movements),<br>5. other solution	Yes or No	Who is responsible for the development measure and what is the timetable	Agree the type of follow-up: phone call or workplace visit	When the development measure is completed
